# Diagnosing alpha-1 antitrypsin deficiency: the first step in precision medicine

**DOI:** 10.12688/f1000research.12399.1

**Published:** 2017-11-27

**Authors:** Craig P. Hersh

**Affiliations:** 1 Channing Division of Network Medicine, Brigham and Women’s Hospital, 181 Longwood Ave., Boston, MA, 02115, USA; 2 Division of Pulmonary and Critical Care Medicine, Brigham and Women’s Hospital, Boston, MA, USA; 3 Harvard Medical School, Boston, MA, USA

**Keywords:** alpha-1 antitrypsin deficiency, diagnosis, testing, COPD

## Abstract

Severe alpha-1 antitrypsin (AAT) deficiency is one of the most common serious genetic diseases in adults of European descent. Individuals with AAT deficiency have a greatly increased risk for emphysema and liver disease. Other manifestations include bronchiectasis, necrotizing panniculitis and granulomatosis with polyangiitis. Despite the frequency and potential severity, AAT deficiency remains under-recognized, and there is often a delay in diagnosis. This review will focus on three recent updates that should serve to encourage testing and diagnosis of AAT deficiency: first, the publication of a randomized clinical trial demonstrating the efficacy of intravenous augmentation therapy in slowing the progression of emphysema in AAT deficiency; second, the mounting evidence showing an increased risk of lung disease in heterozygous PI MZ genotype carriers; last, the recent publication of a clinical practice guideline, outlining diagnosis and management. Though it has been recognized for more than fifty years, AAT deficiency exemplifies the modern paradigm of precision medicine, with a diagnostic test that identifies a genetic subtype of a heterogeneous disease, leading to a targeted treatment.

## Introduction

Severe alpha-1 antitrypsin (AAT) deficiency is one of the most common serious genetic diseases in adults of European descent, with an estimated prevalence of 1 in 1500 to 1 in 3000 ^1, 2^. The AAT protein, the major inhibitor of neutrophil elastase, is encoded by the *SERPINA1* gene on chromosome 14 and secreted from the liver. Severe AAT deficiency is usually due to autosomal recessive inheritance of the PI*Z allele of *SERPINA1*, with the resulting PI ZZ genotype. Severe AAT deficiency is associated with an increased risk of emphysema, especially in cigarette smokers, due to the imbalance between proteases and anti-proteases in the lung tissue. The misfolded Z protein polymerizes in the liver, causing damage to hepatocytes and potentially leading to cirrhosis and hepatocellular carcinoma. Other conditions associated with AAT deficiency include bronchiectasis, necrotizing panniculitis and granulomatosis with polyangiitis.

Despite the high prevalence and potentially serious clinical manifestations, AAT deficiency remains under-recognized, and there is often a delay in diagnosis in symptomatic individuals, as long as five to eight years ^3, 4^. This review will focus on three recent updates that should serve to encourage testing and diagnosis of AAT deficiency, which is necessary to appropriately counsel patients and provide specific therapy. First is the publication of a randomized clinical trial demonstrating the efficacy of intravenous augmentation therapy in slowing the progression of emphysema in patients with chronic obstructive pulmonary disease (COPD) due to AAT deficiency ^5^. Second is the mounting evidence showing an increased risk of airflow obstruction in heterozygous carriers (genotype PI MZ) ^6, 7^. Last is the recent publication of a clinical practice guideline outlining the diagnosis and management of AAT deficiency ^8^. The full spectrum of the genetics, biology, and clinical manifestations of AAT deficiency has been reviewed elsewhere ^9– 12^.

## Clinical trial of augmentation therapy

Augmentation therapy with intravenous infusion of pooled human AAT is available in North America and in several, but not all, European countries. Augmentation therapy was approved based on studies demonstrating biochemical efficacy ^13, 14^. Clinical use has been supported largely from observational and registry studies ^15– 17^. Two small randomized trials suggested a reduction in the loss of lung tissue on chest computed tomography scans ^18, 19^, which was statistically significant when the results were combined in a meta-analysis ^20^.

**Figure 1.**
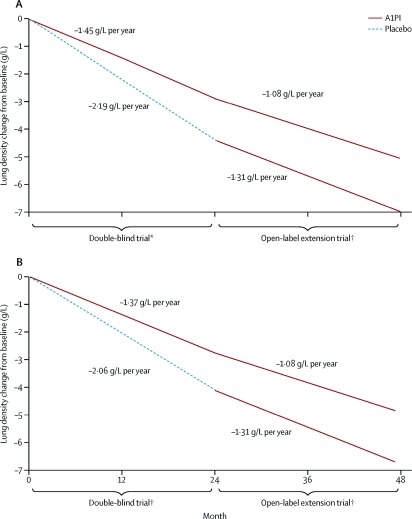
The RAPID trial of augmentation therapy in severe alpha-1 antitrypsin deficiency. Intravenous augmentation therapy slowed the loss of lung density on chest CT scans over four years: ( **A**) all subjects and ( **B**) subjects completing the open-label extension study. Reprinted from 5, with permission from Elsevier.

These studies provided the background for the RAPID trial, a multicenter, double-blind, randomized controlled trial of intravenous AAT augmentation therapy ^5^. Study subjects were former smokers ages 18–65 with severe AAT deficiency (<11 µM) and reduced lung function with a forced expiratory volume in 1 second (FEV _1_) of 35–70% predicted. 95 subjects received AAT infusions at a dose of 60 mg/kg weekly, and 87 subjects received placebo infusions. The primary endpoint was lung density on chest CT scans at total lung capacity (TLC) and functional residual capacity combined, over the two year trial. There was a 29% reduction in the change of combined lung density endpoint between the two arms, which was not statistically significant. However, the change in lung density at TLC was 34% lower in the AAT augmentation group ( *P* = 0.03) ( Figure 1). Lung density measurements at full inspiration (i.e. TLC) are the clinical standard, may be subject to less noise ^21^, and more closely reflect emphysema ^22, 23^. Emphysema progression as an endpoint is more specific to the disease pathology of AAT deficiency than FEV _1_ decline, a more commonly used outcome in COPD clinical trials. Augmentation therapy could be considered as disease-modifying, and was estimated to prolong time to death or lung transplant by almost six years, based on post-hoc extrapolation ^5, 24^. There was no difference in change in lung function, exacerbations, exercise capacity, or quality of life between the treatment and placebo arms.

Following the two-year RAPID randomized trial, subjects from both arms were offered augmentation therapy in a two-year open label extension trial ^24^. Both the group which received AAT augmentation therapy in the randomized trial (early-start) and the group which received placebo (delayed-start) showed the same rate of decline in lung density. However, subjects initially treated with placebo did not recover any of the lost lung density. The extension study showed that the effects of augmentation were sustained over four years and supported earlier treatment initiation.

Patients in RAPID with higher trough serum AAT levels during treatment had less loss of lung density in a post-hoc analysis. A major reason for the increased levels was a greater infused dose of augmentation in patients with higher body weight ^5^. This finding has motivated clinical trials of double dose weekly augmentation therapy ^25^. Other therapies under investigation include inhaled AAT ^26^ and gene therapy ^27^. Based on the RAPID trial, augmentation therapy currently can be recommended in this targeted subgroup of patients with severe AAT deficiency and reduced lung function in a potentially modifiable range (e.g. FEV _1_ 35–70% predicted).

## COPD risk in heterozygous carriers

Starting shortly after the discovery that severe AAT deficiency was associated with COPD ^28^, there has been a debate regarding the risk of lung disease in PI MZ carriers. Most of the literature consisted of small case-control studies, many of which found an association. We performed a meta-analysis, finding an increased risk of COPD with a combined odds ratio (OR) of 2.31 (95% CI 1.60-3.35). The effect was attenuated in studies that adjusted for cigarette smoking (OR 1.61) ^29^. However, population-based studies showed no difference in FEV _1_ between normal genotype (PI MM) and PI MZ individuals. These results were consistent with a small increase in COPD risk in all PI MZ heterozygotes or a larger risk in a subset.

Several pivotal studies have been published subsequently. Sørheim and colleagues examined PI MZ subjects in two large populations: the GenKOLS case-control study and the family-based International COPD Genetics Network (ICGN) ^30^. In both studies, PI MZ heterozygotes had a reduced ratio of FEV _1_ to forced vital capacity (FVC) compared to PI MM (3.5% in GenKOLS, 3.9% in ICGN). In GenKOLS, but not ICGN, PI MZ subjects had more emphysema on quantitative analysis of chest CT scans. There was no difference in COPD case-control status or in airway thickness on chest CT in either study.

Molloy *et al.* performed a family-based study in Ireland ^6^. They identified 51 PI MZ probands with COPD, and enrolled their family members. Comparing 99 PI MM and 89 PI MZ non-index relatives, PI MZ heterozygotes had reduced FEV _1_ and FEV _1_/FVC ratio compared to PI MM. In stratified analyses, this effect was strongest in ever-smokers and was not seen in never smokers. PI MZ was associated with an increased risk of COPD in all subjects (OR 5.18, 95% CI 1.27-21.15).

**Figure 2.**
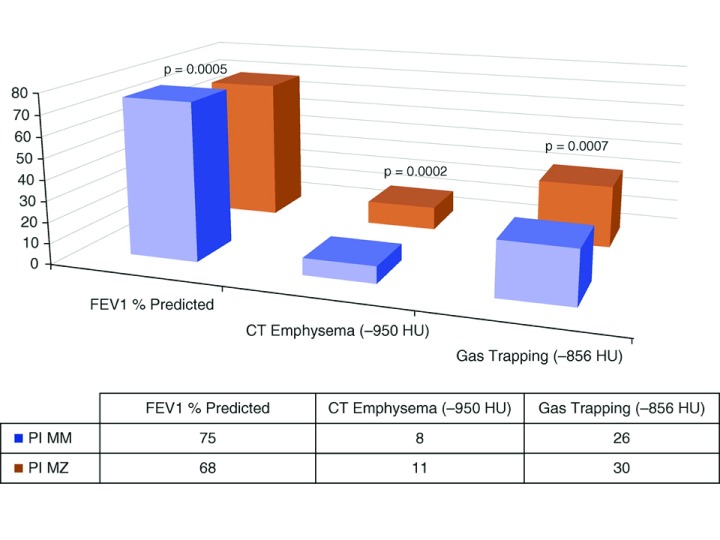
Lung function and chest CT imaging in PI MZ subjects in the COPD Gene Study. Non-Hispanic white subjects with PI MZ genotype had lower lung function and more emphysema and gas trapping on quantitative analysis of chest CT scans. FEV1 = forced expiratory volume in 1 second. HU = Hounsfield units. Reprinted from 7 with permission of the American Thoracic Society. Copyright © 2017 American Thoracic Society. *Annals of the American Thoracic Society* is an official journal of the American Thoracic Society.

Within the COPDGene Study, a large U.S. cohort of current and former smokers with and without COPD, Foreman *et al.* performed genotyping for the *SERPINA1* Z and S alleles ^7^. Out of over 8000 subjects, 261 PI MZ carriers were found, including 22 African Americans. Both white and African American PI MZ subjects had lower FEV _1_ and FEV _1_/FVC ratio compared to PI MM subjects. White PI MZ subjects had an increased risk of COPD (OR 1.42, 95% CI 1.05-1.93) and more emphysema on quantitative analysis of chest CT scans ( Figure 2). African American PI MZ subjects also had increased odds of COPD and increased CT emphysema, though the differences were not statistically significant, likely due to the small sample size.

In an abstract, Ortega *et al*. confirmed the association between PI MZ and reduced lung function and increased emphysema in the Subpopulations and Intermediate Outcome Measures in COPD Study (SPIROMICS) ^31^. An exome genotyping array analysis of over 12,000 subjects also supported the association between PI MZ carriers and COPD ^32^. These studies have clearly demonstrated that PI MZ carriers are at an increased risk for airflow obstruction and COPD, especially in current and former smokers. Molloy *et al*. did not show an increased risk in never smokers ^6^; the other studies did not include enough never smokers to assess their risk.

The mechanisms for COPD risk in PI MZ subjects remain undetermined. Based on serum AAT levels in PI SS individuals, who are not at increased risk of COPD, and PI SZ individuals, who have an increased risk, a protective threshold of 11 μM (approximately 55mg/dl) has been established ^9^. However, serum AAT levels in PI MZ subjects remain above this threshold ^33^.

Although the emphysema in severe AAT deficiency has traditionally been attributed to the lack of inhibition of neutrophil elastase, there is increasing evidence that the Z protein itself may be deleterious in the lung. Accumulated Z-AAT produced in macrophages and lung epithelial cells leads to endoplasmic reticulum (ER) stress, which promotes inflammation, the unfolded protein response, and apoptosis ^34^. Z-AAT in the extracellular space may act as a neutrophil chemoattractant ^35^. A recent study of a Z-AAT transgenic mouse has supported the deleterious effect of the Z protein in the lungs ^36^. These basic mechanisms have been studied in the context of ZZ models, but the Z protein may also have local deleterious effects in the PI MZ lung.

Determining whether the increased COPD risk in PI MZ heterozygotes is due to low levels of AAT (loss of function) or due to the harmful effects of Z-AAT (toxic gain of function) could have therapeutic implications. In the former case, the concept of the protective threshold of 11 μM would have to be revisited, with perhaps different thresholds in current, former, and never smokers. In the latter, PI MZ heterozygotes could be candidates for clinical trials of novel treatments, such as compounds targeting misfolded proteins or ER stress. Regardless of mechanism, identifying PI MZ subjects has implications for risk assessment, family screening, and future precision medicine trials.

## Clinical practice guidelines

The American Thoracic Society (ATS) first published clinical practice guidelines for the evaluation and treatment of patients with AAT deficiency in 1989 ^37^. In collaboration with the European Respiratory Society (ERS), the ATS expanded and updated the guidelines in 2003 ^38^. The Alpha-1 Foundation convened an expert panel, which reviewed the literature since 2002 and produced updated clinical practice guidelines, published in 2016 ^8^.

The guidelines provide recommendations related to testing for AAT deficiency, evaluation of the patient with AAT deficiency, and treatment with intravenous augmentation therapy. The systematic review supports testing in all patients with COPD, as well as patients with other potential manifestations of AAT deficiency, specifically unexplained chronic liver disease, unexplained bronchiectasis, necrotizing panniculitis, and granulomatosis with polyangiitis. Testing is also recommended for first-degree relatives of subjects with severe AAT deficiency. In addition to protein levels, genotyping of at least the S and Z alleles is recommended for initial testing.

Two of the more controversial recommendations relate to evaluation of the newly diagnosed patient with AAT deficiency, namely full pulmonary function testing (spirometry, lung volumes and diffusing capacity) and a baseline chest CT scan ^39^. Chest CT scans are not currently recommended in the initial evaluation of patients with COPD unrelated to AAT deficiency ^40, 41^. The recommendations for serial spirometry to follow lung function and annual liver testing with clinical exam, ultrasound and laboratory studies are less controversial, although there is no clear consensus regarding the age to start liver evaluations ^42^. Serial chest CT scans are not recommended.

Indications for augmentation therapy are largely congruent with the RAPID trial described above. There is a recommendation for discussion of augmentation therapy in patients with milder lung disease (FEV _1_ >65% predicted); declining diffusing capacity or emphysema progression on CT scan may help guide therapy in this situation. Augmentation therapy is not recommended in PI MZ carriers ^43^.

## Conclusions

COPD is a major cause of morbidity and mortality worldwide. Severe AAT deficiency may be responsible for 1–2% of COPD cases in populations of European descent ^44^. Yet AAT deficiency remains under-diagnosed, due to inadequate awareness, lack of referral for appropriate testing, and possibly therapeutic nihilism regarding COPD in general. Three developments in the past several years should provide additional imperatives for diagnosis. Updated clinical practice guidelines provide clear recommendations regarding diagnosis and management ^8^. The RAPID trial has demonstrated the effectiveness of the augmentation therapy for AAT deficiency, with support for earlier initiation ^5^. This fits squarely into the modern paradigm of precision medicine, with a diagnostic test to identify a subtype of a heterogeneous disease, namely COPD, leading to a targeted treatment ^45^. And lastly, the demonstration of an increased risk of COPD in PI MZ carriers may have the broadest public health implications, since there may be over 6 million PI MZ carriers in the U.S. and over 27 million worldwide ^46^. Improved recognition of the role of AAT deficiency in lung and liver disease is crucial for the clinical care of these patients and necessary for the development of novel therapies.

## References

[1] Sveger T: Liver disease in alpha1-antitrypsin deficiency detected by screening of 200,000 infants. *N Engl J Med.* 1976;294(24):1316–21. 10.1056/NEJM197606102942404 1083485

[2] Silverman EK Miletich JP Pierce JA: Alpha-1-antitrypsin deficiency. High prevalence in the St. Louis area determined by direct population screening. *Am Rev Respir Dis.* 1989;140(4):961–6. 10.1164/ajrccm/140.4.961 2679271

[3] Campos MA Wanner A Zhang G: Trends in the diagnosis of symptomatic patients with alpha1-antitrypsin deficiency between 1968 and 2003. *Chest.* 2005;128(3):1179–86. 10.1378/chest.128.3.1179 16162704

[4] Stoller JK Sandhaus RA Turino G: Delay in diagnosis of alpha1-antitrypsin deficiency: a continuing problem. *Chest.* 2005;128(4):1989–94. 10.1378/chest.128.4.1989 16236846

[5] Chapman KR Burdon JG Piitulainen E: Intravenous augmentation treatment and lung density in severe α1 antitrypsin deficiency (RAPID): a randomised, double-blind, placebo-controlled trial. *Lancet.* 2015;386(9991):360–8. 10.1016/S0140-6736(15)60860-1 26026936

[6] Molloy K Hersh CP Morris VB: Clarification of the risk of chronic obstructive pulmonary disease in α1-antitrypsin deficiency PiMZ heterozygotes. *Am J Respir Crit Care Med.* 2014;189(4):419–27. 10.1164/rccm.201311-1984OC 24428606 PMC5955067

[7] Foreman MG Wilson C DeMeo DL: Alpha-1 Antitrypsin PiMZ Genotype Is Associated with Chronic Obstructive Pulmonary Disease in Two Racial Groups. *Ann Am Thorac Soc.* 2017;14(8):1280–7. 10.1513/AnnalsATS.201611-838OC 28380308 PMC5566271

[8] Sandhaus RA Turino G Brantly ML: The Diagnosis and Management of Alpha-1 Antitrypsin Deficiency in the Adult. *Chronic Obstr Pulm Dis.* 2016;3(3):668–82. 10.15326/jcopdf.3.3.2015.0182 28848891 PMC5556762

[9] Silverman EK Sandhaus RA: Clinical practice. Alpha1-antitrypsin deficiency. *N Engl J Med.* 2009;360(26):2749–57. 10.1056/NEJMcp0900449 19553648

[10] Stoller JK Aboussouan LS: A review of α1-antitrypsin deficiency. *Am J Respir Crit Care Med.* 2012;185(3):246–59. 10.1164/rccm.201108-1428CI 21960536

[11] Stockley RA: Alpha1-antitrypsin review. *Clin Chest Med.* 2014;35(1):39–50. 10.1016/j.ccm.2013.10.001 24507836

[12] Marciniak SJ Ordóñez A Dickens JA: New Concepts in Alpha-1 Antitrypsin Deficiency Disease Mechanisms. *Ann Am Thorac Soc.* 2016;13 Suppl 4:S289–96. 10.1513/AnnalsATS.201506-358KV 27564663

[13] Gadek JE Klein HG Holland PV: Replacement therapy of alpha 1-antitrypsin deficiency. Reversal of protease-antiprotease imbalance within the alveolar structures of PiZ subjects. *J Clin Invest.* 1981;68(5):1158–65. 10.1172/JCI110360 7028785 PMC370909

[14] Wewers MD Casolaro MA Sellers SE: Replacement therapy for alpha _1_-antitrypsin deficiency associated with emphysema. *N Engl J Med.* 1987;316(17):1055–62. 10.1056/NEJM198704233161704 3494198

[15] Survival and FEV _1_ decline in individuals with severe deficiency of alpha1-antitrypsin. The Alpha-1-Antitrypsin Deficiency Registry Study Group. *Am J Respir Crit Care Med.* 1998;158(1):49–59. 10.1164/ajrccm.158.1.9712017 9655706

[16] Seersholm N Wencker M Banik N: Does alpha1-antitrypsin augmentation therapy slow the annual decline in FEV1 in patients with severe hereditary alpha1-antitrypsin deficiency? Wissenschaftliche Arbeitsgemeinschaft zur Therapie von Lungenerkrankungen (WATL) alpha1-AT study group. *Eur Respir J.* 1997;10(10):2260–3. 9387950 10.1183/09031936.97.10102260

[17] Wencker M Banik N Buhl R: Long-term treatment of alpha1-antitrypsin deficiency-related pulmonary emphysema with human alpha1-antitrypsin. Wissenschaftliche Arbeitsgemeinschaft zur Therapie von Lungenerkrankungen (WATL)-alpha1-AT-study group. *Eur Respir J.* 1998;11(2):428–33. 9551749 10.1183/09031936.98.11020428

[18] Dirksen A Dijkman JH Madsen F: A randomized clinical trial of alpha(1)-antitrypsin augmentation therapy. *Am J Respir Crit Care Med.* 1999;160(5 Pt 1):1468–72. 10.1164/ajrccm.160.5.9901055 10556107

[19] Dirksen A Piitulainen E Parr DG: Exploring the role of CT densitometry: a randomised study of augmentation therapy in alpha1-antitrypsin deficiency. *Eur Respir J.* 2009;33(6):1345–53. 10.1183/09031936.00159408 19196813

[20] Stockley RA Parr DG Piitulainen E: Therapeutic efficacy of α-1 antitrypsin augmentation therapy on the loss of lung tissue: an integrated analysis of 2 randomised clinical trials using computed tomography densitometry. *Respir Res.* 2010;11(1):136. 10.1186/1465-9921-11-136 20920370 PMC2964614

[21] Parr DG Dirksen A Piitulainen E: Exploring the optimum approach to the use of CT densitometry in a randomised placebo-controlled study of augmentation therapy in alpha 1-antitrypsin deficiency. *Respir Res.* 2009;10(1):75. 10.1186/1465-9921-10-75 19678952 PMC2740846

[22] Washko GR: Diagnostic imaging in COPD. *Semin Respir Crit Care Med.* 2010;31(3):276–85. 10.1055/s-0030-1254068 20496297 PMC4334134

[23] Hersh CP Washko GR Estépar RS: Paired inspiratory-expiratory chest CT scans to assess for small airways disease in COPD. *Respir Res.* 2013;14(1):42. 10.1186/1465-9921-14-42 23566024 PMC3627637

[24] McElvaney NG Burdon J Holmes M: Long-term efficacy and safety of α1 proteinase inhibitor treatment for emphysema caused by severe α1 antitrypsin deficiency: an open-label extension trial (RAPID-OLE). *Lancet Respir Med.* 2017;5(1):51–60. 10.1016/S2213-2600(16)30430-1 27916480

[25] Sorrells S Camprubi S Griffin R: SPARTA clinical trial design: exploring the efficacy and safety of two dose regimens of alpha1-proteinase inhibitor augmentation therapy in alpha1-antitrypsin deficiency. *Respir Med.* 2015;109(4):490–9. 10.1016/j.rmed.2015.01.022 25727857

[26] Campos M Lascano J: Therapeutics: Alpha-1 Antitrypsin Augmentation Therapy. *Methods Mol Biol.* 2017;1639:249–62. 10.1007/978-1-4939-7163-3_25 28752465

[27] Loring HS Flotte TR: Current status of gene therapy for α-1 antitrypsin deficiency. *Expert Opin Biol Ther.* 2015;15(3):329–36. 10.1517/14712598.2015.978854 25363251

[28] Laurell CB Eriksson S: The electrophoretic α _1_-globulin pattern of serum in α _1_-antitrypsin deficiency. 1963. *COPD.* 2013;10 Suppl 1:3–8. 10.3109/15412555.2013.771956 23527532

[29] Hersh CP Dahl M Ly NP: Chronic obstructive pulmonary disease in alpha1-antitrypsin PI MZ heterozygotes: a meta-analysis. *Thorax.* 2004;59(10):843–9. 10.1136/thx.2004.022541 15454649 PMC1746834

[30] Sørheim IC Bakke P Gulsvik A: α _1_-Antitrypsin protease inhibitor MZ heterozygosity is associated with airflow obstruction in two large cohorts. *Chest.* 2010;138(5):1125–32. 10.1378/chest.10-0746 20595457 PMC2972629

[31] Ortega VE Pasha S Ampleford EJ: Rare SERPINA1 Variants are Associated with Lung Function and Emphysema in Non-Hispanic Whites from SPRIOMICS[abstract]. *Am J Respir Crit Care Med.* 2015;191:A3656.

[32] Hobbs BD Parker MM Chen H: Exome Array Analysis Identifies a Common Variant in *IL27* Associated with Chronic Obstructive Pulmonary Disease. *Am J Respir Crit Care Med.* 2016;194(1):48–57. 10.1164/rccm.201510-2053OC 26771213 PMC4960630

[33] Ferrarotti I Thun GA Zorzetto M: Serum levels and genotype distribution of α1-antitrypsin in the general population. *Thorax.* 2012;67(8):669–74. 10.1136/thoraxjnl-2011-201321 22426792

[34] Greene CM McElvaney NG: Protein misfolding and obstructive lung disease. *Proc Am Thorac Soc.* 2010;7(6):346–55. 10.1513/pats.201002-019AW 21030512

[35] Mahadeva R Atkinson C Li Z: Polymers of Z alpha1-antitrypsin co-localize with neutrophils in emphysematous alveoli and are chemotactic *in vivo*. *Am J Pathol.* 2005;166(2):377–86. 10.1016/S0002-9440(10)62261-4 15681822 PMC3278851

[36] Alam S Li Z Atkinson C: Z α1-antitrypsin confers a proinflammatory phenotype that contributes to chronic obstructive pulmonary disease. *Am J Respir Crit Care Med.* 2014;189(8):909–31. 10.1164/rccm.201308-1458OC 24592811 PMC4098095

[37] Guidelines for the approach to the patient with severe hereditary alpha-1-antitrypsin deficiency. American Thoracic Society. *Am Rev Respir Dis.* 1989;140(5):1494–7. 10.1164/ajrccm/140.5.1494 2683912

[38] American Thoracic Society, European Respiratory Society: American Thoracic Society/European Respiratory Society statement: standards for the diagnosis and management of individuals with alpha-1 antitrypsin deficiency. *Am J Respir Crit Care Med.* 2003;168(7):818–900. 10.1164/rccm.168.7.818 14522813

[39] Stoller JK Lacbawan FL Aboussouan LS: Alpha-1 Antitrypsin Deficiency.In: Pagon RA, Adam MP, Ardinger HH, Wallace SE, Amemiya A, Bean LJH, Bird TD, Ledbetter N, Mefford HC, Smith RJH, Stephens K, editors. GeneReviews® [Internet]. Seattle WA: University of Washington, Seattle;2006 Reference Source

[40] Vogelmeier CF Criner GJ Martínez FJ: Global Strategy for the Diagnosis, Management, and Prevention of Chronic Obstructive Lung Disease 2017 Report: GOLD Executive Summary. *Arch Bronconeumol.* 2017;53(3):128–49. 10.1016/j.arbres.2017.02.001 28274597

[41] Yawn BB Thomashaw B Mannino DM: The 2017 Update to the COPD Foundation COPD Pocket Consultant Guide. *Chronic Obstr Pulm Dis.* 2017;4(3):177–85. 10.15326/jcopdf.4.3.2017.0136 28848929 PMC5556909

[42] Nelson DR Teckman J Di Bisceglie AM: Diagnosis and management of patients with α _1_-antitrypsin (A1AT) deficiency. *Clin Gastroenterol Hepatol.* 2012;10(6):575–80. 10.1016/j.cgh.2011.12.028 22200689 PMC3360829

[43] Sandhaus RA Turino G Stocks J: alpha1-Antitrypsin augmentation therapy for PI*MZ heterozygotes: a cautionary note. *Chest.* 2008;134(4):831–4. 10.1378/chest.08-0868 18842915

[44] Lieberman J Winter B Sastre A: Alpha 1-antitrypsin Pi-types in 965 COPD patients. *Chest.* 1986;89(3):370–3. 10.1378/chest.89.3.370 3485034

[45] Jameson JL Longo DL: Precision medicine--personalized, problematic, and promising. *N Engl J Med.* 2015;372(23):2229–34. 10.1056/NEJMsb1503104 26014593

[46] de Serres FJ: Worldwide racial and ethnic distribution of alpha1-antitrypsin deficiency: summary of an analysis of published genetic epidemiologic surveys. *Chest.* 2002;122(5):1818–29. 10.1378/chest.122.5.1818 12426287

